# Functional improvements in patients with lymphangioleiomyomatosis after sirolimus: an observational study

**DOI:** 10.1186/s13023-018-0775-9

**Published:** 2018-02-20

**Authors:** Yongzhong Zhan, Lisha Shen, Wenshuai Xu, Xiuxiu Wu, Weihong Zhang, Jun Wang, Xue Li, Yanli Yang, Xinlun Tian, Kai-Feng Xu

**Affiliations:** 1 Department of Respiratory Medicine, Peking Union Medical College Hospital, Beijing, China; 2 Department of Radiology, Peking Union Medical College Hospital, Beijing, China

**Keywords:** Lymphangioleiomyomatosis, Sirolimus, Pulmonary function, Chylothorax, Adverse events

## Abstract

**Background:**

Sirolimus has been shown to be effective in patients with lymphangioleiomyomatosis (LAM). We wish to summarize our experience using sirolimus and its effectiveness in LAM patients.

**Methods:**

We analyzed data from 98 patients who were diagnosed with definite or probable sporadic LAM based on the European Respiratory Society diagnosis criteria for LAM in 2010 at Peking Union Medical College Hospital and who had received sirolimus during January 2007 to June 2015. The data before and after the initiation of sirolimus therapy included pulmonary function tests, arterial blood gas analysis, 6-min walking distance (6MWD), size of chylous effusion and renal angiomyolipomas (AML), St. George’s Respiratory Questionnaires (SGRQ) and vascular endothelial growth factor-D (VEGF-D) levels. Serum levels of sirolimus and adverse events were collected.

**Results:**

Median follow-up was 2.5 years. Most patients had forced expiratory volume in 1 s (FEV_1_) values less than 70% predicted or symptomatic chylothorax. The mean changes before and after the initiation of sirolimus were − 31.12 ± 30.78 mL/month and 16.11 ± 36.00 mL/month (*n* = 18,*p* = 0.002) for FEV_1_ change, and − 0.55 ± 0.60 mmHg/month and 0.30 ± 1.19 mmHg/month (*n* = 17, *p* = 0.018) for P_a_O_2_ change. 6MWD improved from 358.8 ± 114.4 m to 415.6 ± 118.6 m (*n* = 46, *p* = 0.004) and SGRQ total score from 57.2 ± 21.0 to 47.5 ± 22.8 (*n* = 50, *p* < 0.001). The median VEGF-D concentration decreased to 1609.4 pg/mL from 3075.6 pg/mL after sirolimus therapy (*n* = 41, *p* < 0.001). Patients with sirolimus trough levels of 5–9.9 ng/mL had an increase in FEV_1_ (*p* < 0.05). Sixty-five percent of patients (13/20) had almost complete resolution of chylous effusions. The most frequent adverse events were mouth ulcers, menstrual disorder, hyperlipidemia and acneiform rash, all were mild.

**Conclusion:**

Long-term use of sirolimus is safe in patients with LAM. LAM patients with FEV_1_ less than 70% predicted and symptomatic chylothorax are suitable for receiving sirolimus therapy. The maintaining serum trough levels of sirolimus are recommended between 5 to 9.99 ng/mL.

## Background

Lymphangioleiomyomatosis (LAM) is a rare multisystem neoplastic disorder that mostly afflicts women and primarily affects the lung and kidney [1]. The prevalence of sporadic LAM varies, ranging from 1 to 9 per million women in the general population and 30–40% of women with tuberous sclerosis complex (TSC) [2, 3]. Patients with LAM suffer from worsening dyspnea and an increasing number of cysts by computed tomography (CT) detection. Forced expiratory volume in 1 s (FEV_1_), one of the most important pulmonary function measurements, declines at a rate of 75 to 134 mL per year [4, 5]. The management of LAM, however, focuses on observation and supportive therapy. Few effective medications have been identified to slow disease progression.

Loss of TSC2 gene expression plays a central role in the pathogenesis of LAM [6]. The TSC1/TSC2 complex persistently activates the mammalian target of rapamycin (mTOR) signaling pathway, which regulates cellular metabolism, growth and survival. Sirolimus (also called rapamycin), an mTOR inhibitor, maintains mTOR downstream signals and multiple cellular functions at appropriate levels [7]. Sirolimus has been shown to be efficacious in sporadic LAM and TSC-associated patients regarding lung function, lymphatic disease and renal angiomyolipomas in previous studies, including the Multicenter International Lymphangioleiomyomatosis Efficacy and Safety of Sirolimus (MILES) trial [5, 8–14]. These studies have provided evidence for using sirolimus in LAM patients with moderately severe lung disease, chylothorax, chylous ascites or renal angiomyolipomas. In Peking Union Medical College Hospital (PUMCH), sirolimus has been used in LAM patients since 2007, and much data have been accumulated during this period. This study was aimed at summarizing our experience and providing more evidence regarding the safety, indication, timing and dose of sirolimus.

## Methods

### Study population

We reviewed records from patients at PUMCH between January 2007 and June 2015. The patients were included if they 1) were diagnosed with definite or probable LAM, based on the European Respiratory Society (ERS) guidelines in 2010 [15] and 2) received sirolimus treatment for at least 12 months. Patients with TSC-associated LAM were excluded in this analysis. The study was part of the LAM registry study of PUMCH. The protocol was approved by the Ethical Committee of PUMCH (S-379). All subjects included in this study signed informed consent documents.

### Medication use

Most patients included in this study followed common rules to adjust their sirolimus dose. The initial dose was 2 mg per day if the patient weighed ≥50 kg and 1 mg per day if the patient weighed < 50 kg. The patients were advised to perform a concentration test one month after the initiation of sirolimus. A serum level of 5 to 9.9 ng/mL sirolimus was the target range. Patients with a sirolimus concentration ≥ 10 ng/mL were recommended to reduce the dose by half. For patients with serum levels < 5 ng/mL, if the clinical symptoms were improving according to a physician’s evaluation, the initial dose was continued; if not, the dose of sirolimus was adjusted. After adjustment of the sirolimus dose, the concentration test and symptom evaluation were repeated every 1 to 3 months. The dose of sirolimus was also adjusted due to adverse events or cost burden.

### Study design

The records of the included patients were carefully reviewed. We collected the following data: pulmonary function tests, arterial blood gas analysis at rest (on room air), six-minute walking distance (6MWD), St. George’s Respiratory Questionnaire (SGRQ), vascular endothelial growth factor-D (VEGF-D), and response rate of the chylothorax and presence of renal angiomyolipomas by radiological evaluation. We defined baseline data as those collected within 30 days of sirolimus initiation, and additional data were collected 6 months (±3 months), 12 months (±3 months) and ≥15 months after sirolimus initiation. The rate of change in each parameter per month was calculated with data from the most remote two points and time duration. The response of the chylothorax was defined as almost no effusion present on any kind of imaging examination, including chest X-ray, CT and ultrasound, after 6 months without any other interventional or surgical procedures. Adverse events were defined as symptoms that appeared after the therapy was initiated and that lasted for at least 2 days or resulted in abnormal laboratory findings.

Pulmonary function tests were performed according to the ATS/ERS Task Force Standardization of Lung Function Testing in 2005 [16]. The six-minute walking tests were evaluated based on ATS Guidelines for the six-minute walk test [17]. The SGRQs were completed by patients according to the provided instructions [18]. The chylothorax was evaluated by chest X-ray or CT. Renal angiomyolipoma size was evaluated by CT (plain or contrast-enhanced). All imaging was done in a radiology department and interpreted by both radiologists and respiratory physicians. Serum VEGF-D levels were tested by enzyme-linked immunosorbent assay (R&D Systems).

### Statistical analysis

The normality of the data was analyzed by Kolmogorov-Smirnov test. Data in normal distribution were reported as means±SD and data in non-normal distribution were reported as median (Q1, Q3). Paired and unpaired *T* tests were used to compare the differences before and after treatment. All reported *p* values are two-sided. *p* values of less than 0.05 were considered statistically significant. All analyses were performed using SPSS software, version 20.0 (IBM, USA).

## Results

### Patient characteristics

From January 2010 to June 2015, 231 patients were diagnosed with definite or probable sporadic LAM in PUMCH. Of these, 98 patients were enrolled into this study according to the inclusion and exclusion criteria. The number of definite LAM patients was 70, and the number of probable LAM patients was 28. The clinical characteristics of these patients are shown in Table 1. The most frequent reasons for starting sirolimus therapy was FEV_1_ less than 70% predicted. Four patients started sirolimus for early treatment; three of these patients had rapidly declining pulmonary function, which presented as FEV_1_ decreasing by more than 90 mL per year but still within the normal range. Another patient had normal and stable pulmonary function. Some patients (3.1%) were treated with sirolimus for unknown reasons because of insufficient data. Median follow-up was 2.5 (2.0, 4.0) years.

**Table 1:** Baseline demographic and clinical characteristics of patients with lymphangioleiomyomatosis

Characteristics	Numbers (%)
Patients numbers	98
Age-year	21–62
Gender	
Female	98 (100)
Race	
Asian	98 (100)
Diagnosis	
Definite LAM	78 (79.6)
Probable LAM	20 (20.4)
Clinical features	
Postmenopause	5 (5.1)
Previous pregnancy	76 (77.6)
Former smoker	0 (0)
Renal angiomyolipomas	19 (19.4)
Retroperitoneal angiomyolipomas	2 (2.0)
History of pneumothorax	42 (42.8)
History of chylothorax	35 (35.7)
Pulmonary-functions^a^	
FEV_1_ (L)	1.51 ± 0.74
FEV_1_/pred (%)	49.03 ± 24.64
FVC (L)	2.55 ± 0.74
FVC/pred (%)	76.31 ± 22.99
FEV_1_/FVC (%)	51.22 ± 16.53
PaO_2_ on room air (mmHg)	65.26 ± 11.39
Reasons for sirolimus-No. (%)	
FEV_1_ less than 70% predicted	65 (66.3)
Chylothorax or other lymphatic manifestations^b^	22 (22.4)
Renal angiomyolipoma	4 (4.1)
Early treatment^c^	4 (4.1)
Unknown reasons^d^	3 (3.1)
Types of sirolimus used^e^	
Rapamune	76 (77.6)
Yixinke	21 (21.4)
Saimosi	1 (1.0)

*Abbreviations*: *LAM* lymphangioleiomyomatosis, *FEV*_*1*_ forced expiratory volume in 1 s

^a^Sample size for spirometry and blood gas analysis was 71 and 81 respectively

^b^Including measurable lymphangiomyomas, lymphangiectasis and lymphedema

^c^Including 3 patients with FEV_1_ annual decreases of more than 90 mL and 1 patient with normal and stable FEV_1_

^d^Reason for sirolimus could not be identified because of insufficient data

^e^Rapamune, Pfizer Pharmaceuticals Ltd.; Yixinke, North China Pharmaceuticals Co., Ltd.; Saimosi, Hangzhou Sino-American Pharmaceuticals Ltd

### Sirolimus improves FEV_1_ of pulmonary function tests

**Fig. 1:**
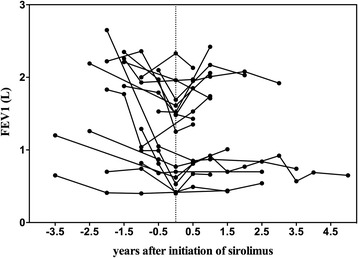
Change in FEV_1_ before and after sirolimus in paired groups. Zero on the horizontal axis indicates the day on which sirolimus therapy was initiated; negative and positive numbers indicate years before and after the initiation of sirolimus administration, respectively. Abbreviations: FEV_1_, forced expiratory volume in 1 s

The rates of change in pulmonary function variables before sirolimus could be obtained for 38 patients (− 22.20±26.07 ml/month), and the rates of change after sirolimus could be obtained for 34 patients (12.23±31.54 ml/month). In 18 patients, the change rates both before and after sirolimus treatment were obtained. In paired comparisons, the mean monthly changes of FEV_1_ were − 31.12 ± 30.78 mL and 16.11 ± 36.00 mL, respectively (*p* = 0.002) (Table 2, Fig. 1). FEV_1_% of predicted value, FVC% of predicted value, FEV_1_/FVC and DL_CO_% of predicted value were also improved significantly in paired comparisons (Table 2). Unpaired comparisons before sirolimus and after sirolimus showed similar results except insignificant changes of DL_CO_ (data not shown).

**Table 2:** Effects of sirolimus on rates of change in pulmonary function and arterial blood gas

Variable	Change per month (Paired)		
	Before sirolimus	After sirolimus	*P* value
Pulmonary Function	(*N* = 18)		
FEV_1_ (mL)	−31.12 ± 30.78	16.11 ± 36.00	0.002
FEV_1_/pred (%)	−0.95 ± 0.82	0.30 ± 0.89	< 0.001
FVC/pred (%)	−1.13 ± 0.93	0.71 ± 1.36	0.001
FEV_1_/FVC (%)	−0.82 ± 1.36	0.78 ± 2.16	0.019
RV/pred (%)	2.50 ± 9.01	0.16 ± 8.30	0.497
TLC/pred (%)	1.02 ± 4.49	1.15 ± 6.20	0.953
RV/TLC (%)	0.78 ± 2.38	0.41 ± 2.58	0.688
DL_CO_/pred (%)	−0.72 ± 1.34	1.10 ± 2.28	0.043
Arterial Blood Gas	(*N* = 17)		
P_a_O_2_ (mmHg)	−0.55 ± 0.60	0.30 ± 1.19	0.018
P_a_CO_2_ (mmHg)	0.005 ± 0.327	0.058 ± 0.194	0.170
P_(A-a)_O_2_ (mmHg)	0.31 ± 0.92	−0.75 ± 1.28	0.037

*Abbreviations*: *FEV*_*1*_ forced expiratory volume in 1 s, *FVC* forced vital capacity, *RV* residual volume, *TLC* total lung capacity, *DL*_*CO*_ diffusing capacity for carbon monoxide, *P*_*a*_*O*_*2*_ partial pressure of oxygen in arterial blood, *P*_*a*_*CO*_*2*_ partial pressure of carbon dioxide in arterial blood, *P*_*(A-a)*_*O*_*2*_ alveolar-arterial oxygen gradient

Data are presented as mean ± SD

The FEV_1_ value, FEV_1_% of predicted value, FVC% of predicted value for the patients were significantly increased after 6 months and ≥15 months on sirolimus relative to the baseline levels (*p* < 0.05). These variables also increased after 12 months of sirolimus therapy, but these changes were not statistically significant (Table 3). Some patients had FEV_1_ values above their baseline values after 6 months (85.7%, 12/14), whereas the remaining patients (14.3%, 2/14) showed slight decreases relative to their baseline FEV_1_ values. All nine patients had FEV_1_ values greater than (8/9) or equal to (1/9) baseline levels after more than 15 months on sirolimus.

**Table 3:** Paired comparisons of functional tests from baseline to various time points after sirolimus

Variable	6 month			12 months			≥15 months		
	Baseline	After sirolimus	*P* value	Baseline	After sirolimus	*P* value	Baseline	After sirolimus	*P* value
Pulmonary function	(*N* = 14)			(*N* = 9)			(*N* = 9)		
FEV_1_ (L)	1.00 ± 0.42	1.21 ± 0.47	0.002	1.56 ± 0.85	1.70 ± 0.66	0.272	0.99 ± 0.45	1.20 ± 0.59	0.007
FEV_1_/pred (%)	36.7 ± 16.8	44.5 ± 18.1	0.003	53.1 ± 29.0	58.5 ± 22.5	0.237	34.7 ± 14.2	43.3 ± 19.3	0.002
FVC/pred (%)	68.5 ± 26.0	83.0 ± 25.0	0.001	72.4 ± 20.7	82.6 ± 12.5	0.059	62.8 ± 16.6	84.7 ± 17.3	0.007
Arterial blood gas	(*N* = 21)			(*N* = 16)			(*N* = 14)		
P_a_O_2_ (mmHg)	63.8 ± 9.2	70.1 ± 14.0	0.017	65.6 ± 13.0	67.9 ± 15.0	0.595	62.1 ± 8.7	71.4 ± 11.2	0.001
P_a_CO_2_ (mmHg)	34.2 ± 3.1	34.5 ± 3.5	0.980	32.9 ± 3.7	31.6 ± 7.3	0.970	33.3 ± 4.3	34.5 ± 4.3	0.238
P_(A-a)_O_2_ (mmHg)	43.1 ± 10.1	43.1 ± 10.1	0.293	45.1 ± 12.7	39.1 ± 16.6	0.083	48.4 ± 10.5	39.5 ± 15.0	0.011
6-min walk test	(*N* = 22)			(*N* = 18)			(*N* = 13)		
6MWD (m)	352.5 ± 143.5	442.2 ± 103.9	< 0.001	367.3 ± 135.5	448.7 ± 100.9	0.001	320.7 ± 138.0	423.3 ± 127.1	0.016
St. George’s Respiratory Questionnaire	(*N* = 27)			(*N* = 20)			(*N* = 16)		
Symptoms score	52.7 ± 23.7	37.6 ± 25.4	0.001	48.1 ± 24.8	32.0 ± 26.7	< 0.001	51.4 ± 21.1	29.4 ± 17.1	< 0.001
Activity score	68.1 ± 26.0	56.1 ± 25.4	< 0.001	63.8 ± 25.3	51.6 ± 24.7	0.009	70.9 ± 23.2	53.7 ± 25.0	0.001
Impacts score	53.1 ± 27.0	37.2 ± 27.0	< 0.001	48.4 ± 25.3	32.5 ± 22.9	0.001	55.8 ± 23.7	33.2 ± 21.7	0.001
Total score	57.6 ± 24.8	43.0 ± 23.7	< 0.001	53.2 ± 23.9	38.2 ± 23.0	< 0.001	59.8 ± 21.9	38.8 ± 19.6	< 0.001

*Abbreviations*: *FEV*_*1*_ forced expiratory volume in 1 s, *FVC* forced vital capacity, *P*_*a*_*O*_*2*_ partial pressure of oxygen in arterial blood, *P*_*a*_*CO*_*2*_ partial pressure of carbon dioxide in arterial blood, *P*_*(A-a)*_*O*_*2*_ alveolar-arterial oxygen gradient, *6MWD* six-minute walking distance

Data are presented as means±SD

Four patients were considered as having received early treatment. One patient was in a normal and stable state of lung function and did not have any other obvious manifestations when she started sirolimus therapy. This was a 56-year-old woman who maintained normal pulmonary function test results three years after initiating sirolimus and seven years after receiving a diagnosis of LAM. An additional three patients also had normal pulmonary function tests but showed trends towards rapidly declining FEV_1_ (> 90 mL per year). Two of these patients were able to reach stable lung function during sirolimus therapy. The FEV_1_ of the remaining patient decreased by 130 mL per year before sirolimus and 250 mL per year after sirolimus.

### Sirolimus improves the oxygen level

Sirolimus significantly improved the arterial oxygen levels and alveolar arterial oxygen gradient according to the rates of change (Table 2) and absolute values at different time points after the therapy (Table 3). When compared with baseline measurements, 71.4% (15/21), 75% (12/16) and 85.7% (12/14) of the patients had higher P_a_O_2_ after 6, 12 and ≥15 months on sirolimus. In total, P_a_O_2_ was 5 mmHg higher on average after sirolimus treatment than at baseline.

### Sirolimus improves the 6-min walking distance (6MWD)

In a paired group analysis (*n* = 46), the 6MWD was 358.8 ± 114.4 m before sirolimus and 415.6 ± 118.6 m after sirolimus (*p* < 0.05). The mean increase was 56.8 m. The 6MWD results after 6 months, 12 months and ≥15 months on sirolimus increased significantly compared with baseline data (*p* < 0.05).

### Sirolimus improves the resolution of chylothorax

Twenty patients started sirolimus therapy because of symptomatic chylothorax, and two of these patients were complicated with chylous ascites. Chylous effusions had been present 1.72 ± 2.17 years before the initiation of sirolimus therapy. Before sirolimus therapy, all patients had undergone thoracentesis, and 6 patients had required chest tube drainage. Surgical intervention had been performed in 7 of the 20 patients, including pleurodesis in 3 patients, lymphatic venous anastomosis in 3 patients, ligation of the thoracic duct in 1 patient and lysis of thoracic duct adhesions in 1 patient. One of these patients had undergone both pleurodesis and lymphatic venous anastomosis. These interventions, however, did not prevent the recurrence of pleural effusions in any patient. During sirolimus therapy, 13 patients had almost complete resolution of their pleural effusions, and the response rate reached 65%. Two patients still experienced chylous effusions during sirolimus therapy and required thoracentesis. The results of another 5 patients could not be found in our database.

Two patients started sirolimus therapy for severe lymphatic manifestations and experienced relief during the therapy. One patient was a 34-year-old woman who had her retroperitoneal lymphangiomyoma resected in 2006 and found it recurrent and progressing in size (the maximum diameter was 2.9 cm) in 2012. During sirolimus therapy (2012 to present), the mass decreased and disappeared on abdominal CT in 2016. The other patient, a 44-year-old woman, had multiple retroperitoneal lymphangiomyomas and lymphangiectasis and severe lymphedema in her left lower extremity without chylous effusions or ascites in 2012 and initiated sirolimus at that time. She also experienced relief from the lymphatic manifestations during follow-up.

### Effects of sirolimus on angiomyolipomas

Of the 19 patients with renal angiomyolipomas, 11 had angiomyolipomas diameter more than 1 cm. We had follow-up data to 2015 in 6 patients and 5 of them had a decrease in tumor diameter after sirolimus therapy. Another one had her mass resected before initiating sirolimus and did not got any recurrence during sirolimus therapy, for 14 months in data. 2 patients had retroperitoneal angiomyolipomas in their history. One of them had it resected before sirolimus therapy and did not have any recurrence during sirolimus therapy, for 18 months in data. Another one did not undergo follow-up abdominal CT.

### Sirolimus improves the quality of life (SGRQ score)

In paired groups (*n* = 50), symptom scores, activity scores, impact scores and total scores of SGRQ after sirolimus therapy all exhibited significant decrease from the baseline value of 51.2±21.8, 67.1±21.0, 52.7±23.4 and 57.2±21.0 to the after treatment value of 41.0±25.1, 59.9±23.5, 42.3±25.0, and 47.5±22.8 respectively (*p* < 0.05). The mean decrease in SGRQ total scores was 9.7. SGRQ scores at 6 months, 12 months and 15 months after initiation of sirolimus therapy all declined significantly compared with baseline data (*p* < 0.05) (Table 3).

### Effects of sirolimus on serum VEGF-D concentrations

The median serum VEGF-D concentration was 3075.6 pg/mL (2406.5 pg/mL, 4359.6 pg/mL) before sirolimus and 1609.4 pg/mL (1162.1 pg/mL, 2457.1 pg/mL) after sirolimus (*n* = 41, *p* < 0.05). Baseline VEGF-D levels may predict treatment response of oxygen levels. When patients were divided to two groups based on P_a_O_2_ changes after treatment (< 0.3 mmHg/month, *n* = 20 and ≥ 0.3 mmHg/month, n = 20), significant difference of baseline VEGF-D levels, 2665.7 (1723.4, 3144.9) pg/ml and 4589.5 (2140.0, 3475.5) pg/ml respectively, was found (*p* < 0.05). There was no difference of baseline VEGF-D levels in two groups based on FEV_1_ changes after treatment (< 4 ml/month, *n* = 14 and ≥ 4 ml/months, *n* = 16).

### Serum levels of sirolimus

The serum levels of sirolimus were tested at PUMCH in 59 patients. Most patients (72.9%) maintained sirolimus levels between 5 to 9.9 ng/mL. Some patients (20.3%) had sirolimus levels under 5 ng/mL but all above 3 ng/mL. Patients with sirolimus trough levels 5–9.9 ng/mL had significant increases in FEV_1_ (*p* < 0.05). Patients with low-dose sirolimus (trough levels 3–4.9 ng/mL) and relatively high-dose (trough levels 10–14.9 ng/mL) showed only a tendency to an increase in FEV_1_, but the increase was not statistically significant (*p* > 0.05).

### Adverse events potentially related to sirolimus

The most common adverse events were mouth ulcers, menstrual disorder, hyperlipidemia and acneiform rash (Table 4). Almost all adverse events were mild. Only three patients discontinued sirolimus due to severe adverse events, which included elevated liver enzymes, lung infection and fever. All three patients initiated the therapy again after 2 to 6 months and were free of severe adverse events.

**Table 4:** Adverse events in patients with sirolimus^a^

Adverse events	Number of patients	Percentage (%)
Mouth ulcers	43	43.9
Menstrual abnormality	26	26.5
Hyperlipidemia	15	15.3
Acneiform rash	15	15.3
Diarrhea	2	2.0
Fever	2	2.0
Peripheral edema	2	2.0
Gingival hyperplasia	2	2.0
Infections	1	1.0
Hypertension	1	1.0
Non-productive cough	1	1.0
Nasal allergy	1	1.0
Low back pain	1	1.0
Chest pain	1	1.0
Alopecia	1	1.0
Toothache	1	1.0
Arthralgia (finger)	1	1.0
Dizziness	1	1.0
Palpitation	1	1.0
Elevated alanine aminotransferase	1	1.0

^a^Three patients stopped sirolimus because of severe adverse effects, which were elevated liver alanine aminotransferase, lung infection and fever. All of them initiated the therapy again after 2 to 6 months and were free of severe adverse events

## Discussion

In the present study, LAM patients, most of whom had damaged pulmonary function and lymphatic manifestations, benefited from sirolimus therapy without severe adverse events. Sirolimus stabilized pulmonary function and arterial partial oxygen pressure, improved quality of life and exercise tolerance, and induced remission of lymphatic manifestations, especially chylothorax. Sirolimus also reduced serum levels of the disease severity associated biomarker VEGF-D.

For functional evaluation, pulmonary function variables are the most direct way to determine the efficacy of sirolimus. Before sirolimus therapy, the patients were experiencing losses in FEV_1_ of 266.40 mL per year, which was 8.8 times that of the annual loss in healthy people [19]. After sirolimus treatment, FEV_1_ was remarkably improved to adding 146.76 ml per year that was higher than previous reports [5, 8, 10].

In addition to the patients with severely impaired lung function, we also looked on those patients who had relatively normal lung function. An annual loss of 90 mL or more in the FEV_1_ value was considered rapidly declining as this is three-fold greater than the normal rate of annual loss in healthy people [19, 20]. Two patients out of three eliminated the FEV_1_ rapid loss during sirolimus therapy, which reminded us to pay more attention to the declining rate rather than the absolute value of lung function. Another patient who started sirolimus with normal and stable lung function and had persistently stable FEV_1_ during sirolimus provided physicians with a choice of initiating sirolimus at the time of diagnosis so that the patient’s lung function could be stabilized as early as possible.

Patients evaluated after 15 months of sirolimus therapy still showed stabilization of lung function, which indicated that sirolimus had a sustained effect on the LAM patients. The importance of the observed changes in lung function was further supported by arterial blood gas analysis, 6MWD and SGRQ scores. These results were consistent with those from two long-term observational studies [8, 11]. P_a_O_2_ is a comprehensive manifestation of ventilation and gas exchange function, both of which are damaged in LAM patients. Actually, the positive change in P_a_O_2_ was in accordance with other variables associated with lung function, indicating that it was also an effective evaluation measurement. The 6MWD was stabilized but not improved after sirolimus in the MILES trial [5]. In another observation of only 5 patients, 6MWD significantly improved after sirolimus [21]. The patients in our study showed a significant increase of 57 m, which demonstrated the sirolimus-related effects on exercise tolerance. SGRQ was used in our study to evaluate the quality of life, representing overall status of health of patients. Our results suggested SGRQ was sensitive showing treatment response after sirolimus. In our previous study, we found SGRQ is correlated with Borg scale of breathlessness, 6MWD, oxygen and pulmonary functions in LAM patients [22]. Quality of life improvement after sirolimus was also reported in MILES study evaluated with EuroQoL visual-analogue scale and Functional Performance Inventory [5]. MILES study also confirmed the correlations of SGRQ and FEV_1_, DL_CO_, and 6MWD [23]. Exercise capacity and health-related quality of life are important outcomes in LAM patients.

At least 65% of the patients (13/20) who had symptomatic chylothorax had favorable responses to sirolimus in our study. Taveira-DaSilva reported that all 12 patients with chylous effusions experienced resolution of this condition, and 9 of these patients had complete resolution [10]. Another study showed that chylothorax resolved completely within 1 to 5 months of sirolimus therapy in 6 of 7 cases. Several case reports have also supported the effectiveness of sirolimus in the remission of chylous effusions [24–28].

With respect to dosing, the sirolimus dose was generally adjusted to maintain a serum trough level between 5 and 15 ng/mL [5, 10, 11, 29]. A Japanese and a recent United Kingdom study suggested that low-dose sirolimus, which resulted in serum trough levels lower than 5 ng/mL, was also effective in stabilizing lung function and resolving chylothorax [9, 30]. In our study, however, patients with low-dose sirolimus (trough levels 3–4.9 ng/mL) showed increases in FEV_1_, but this change was not statistically significant (*p* > 0.05). We should pay attention to the fact that the number of patients was small (*n* = 5). Interestingly, adjusting sirolimus dose to serum trough levels being 5–9.9 ng/mL, but not 5–15 ng/mL, was effective enough to improve lung function in LAM patients.

This study described our experience of using sirolimus in a real practice with LAM patients. There are several limitations. The follow-up evaluations were not comprehensive due to irregular follow-up, unwillingness to be evaluated, or inability to be evaluated due to health status or medical costs. Second, the sample sizes in some subgroup analyses were small. We are now performing an updated version of a registry study of LAM using a nation-wide sample from China (LAM-China, ClinicalTrials.gov# 03193892). Patients in the LAM-China study will be evaluated annually.

## Conclusion

Sirolimus is effective in stabilizing or improving pulmonary function, blood oxygen levels, exercise capacity, and quality of life in patients with LAM. Sirolimus is effective in the treatment of LAM-related chylothorax. Sirolimus also decreases the VEGF-D levels, a biomarker of LAM disease severity. Sirolimus is safe in long-term use with a median follow-up of 2.5 years. A dose of sirolimus between 5 and 9.9 ng/mL is appropriate.

## Abbreviations

6MWD: 6-min walking distance
AML: Angiomyolipomas
DL_CO_: Diffusing capacity for carbon monoxide
ERS: European Respiratory Society
FEV_1_: Forced expiratory volume in 1 s
FVC: Forced vital capacity
LAM: Lymphangioleiomyomatosis
MILES: Multicenter International Lymphangioleiomyomatosis Efficacy and Safety of Sirolimus
mTOR: Mammalian target of rapamycin
P_(A-a)_O_2_: Alveolar-arterial oxygen gradient
P_a_CO_2_: Partial pressure of carbon dioxide in arterial blood
P_a_O_2_: Partial pressure of oxygen in arterial blood
PUMCH: Peking Union Medical College Hospital
RV: Residual volume
SGRQ: St. George’s Respiratory Questionnaires
TLC: Total lung capacity
TSC: Tuberous sclerosis complex
VEGF-D: Vascular endothelial growth factor-D
